# P-824. Successful Diagnostic Stewardship Intervention in Reducing Unnecessary Stool Testing for C. difficile in a Veterans Affairs Hospital

**DOI:** 10.1093/ofid/ofaf695.1032

**Published:** 2026-01-11

**Authors:** Lisa Bailey, Monique Thorne, Debra Noland, Jessica Schildkraut, Florence M Ford, Sabrina Mohsin, George Psevdos

**Affiliations:** Northport VAMC, Northport, New York; Northport VAMC, Northport, New York; Northport VAMC, Northport, New York; Northport VAMC, Northport, New York; Northport VA, Northport, New York; Stony Brook University Hospital, Branchburg, NJ; Northport VA Medical Center, Northport, New York

## Abstract

**Background:**

*Clostridioides difficile* (C diff) is a highly contagious bacterium that causes diarrhea and colitis in people who’ve recently completed antibiotic therapy. While early diagnosis can ensure timely treatment, inappropriate testing may identify patients who are harmlessly colonized rather than having an acute disease. The Infectious Diseases Society of America recommends targeted testing for patients with clear symptoms/presentation associated with *C. difficile* infection (CDI) avoiding universal C diff testing to all patients with gastrointestinal (GI) symptoms, thus preventing needless treatment. In 2022 the infection control team at Northport Veterans Affairs Medical Center decided to use a template note-order due to increase of numbers of C diff tests that were deemed colonization. We assessed the performance of a templated C diff order in our electronic medical record system (EMR) with the aim to reduce unneeded testing

**Methods:**

A templated order was created in our EMR called computerized patient record system (CPRS) and implemented in late 2022. A provider, resident in training or attending physician must complete this note first to generate an order for stool C diff testing. We compared C diff testing rates from 2019-2022 to 2023-2024. Figure 1 depicts the testing algorithm and figure 2 an example of the template note. Also, the microbiology lab was instructed to hide the result of the C diff PCR result which is part of the GI PCR panel. Only GDH/C-diff toxin assay can be ordered through the template note

**Results:**

Table 1 depicts the number of C diff tests, CDI, and colonization. Comparing the study years there was a decrease in the median tests ordered 250 vs. 187, P:0015. In 2022 we noted a spike in inappropriate C diff testing with 13 positive results deemed as colonization. Education by infection preventionists was provided to our acute care and nursing home teams on rationale for the template note. In 2016 our antimicrobial stewardship program was initiated and since its start there has been a continued success in reducing CDI cases in our hospital, see figure 3

**Conclusion:**

The template EMR order note led to a decrease in C diff testing overall and with a decrease in positive colonization tests. C diff diagnostic stewardship remains a critical component in infection prevention and control efforts

**Disclosures:**

All Authors: No reported disclosures

